# Lessons Learned from Pompe Disease Newborn Screening and Follow-up

**DOI:** 10.3390/ijns6010011

**Published:** 2020-02-14

**Authors:** Tracy L. Klug, Lori B. Swartz, Jon Washburn, Candice Brannen, Jami L. Kiesling

**Affiliations:** 1 Missouri Department of Health and Senior Services, P.O. Box 570, Jefferson City, MO 65102-0570, USA; 2 Baebies, Inc., P.O. Box 14403, Durham, NC 27709, USA

**Keywords:** Pompe disease, newborn screening, follow-up, pseudodeficiency

## Abstract

In 2015, Pompe disease became the first lysosomal storage disorder to be recommended for universal newborn screening by the Secretary of the U.S. Department of Health and Human Services. Newborn screening for Pompe has been implemented in 20 states and several countries across the world. The rates of later-onset disease phenotypes for Pompe and pseudodeficiency alleles are higher than initially anticipated, and these factors must be considered during Pompe disease newborn screening. This report presents an overview of six years of data from the Missouri State Public Health Laboratory for Pompe disease newborn screening and follow-up.

## 1. Introduction

Pompe disease, also called glycogen storage disease type II, is a rare genetic disorder in which variants in the glucosidase alpha acid gene (gene abbreviation: *GAA*) result in low levels of acid alpha-glucosidase enzyme (enzyme abbreviation: GAA) and consequent accumulation of glycogen in various tissues of the body [1,2]. The build-up of glycogen damages muscles throughout the body, most notably the heart and skeletal muscle, and leads to general muscle weakness, breathing problems, and feeding difficulties. The onset and severity of disease symptoms vary widely based on the precise *GAA* variant(s) inherited [3]. The most severe phenotype, the classical infantile form, is clinically apparent in the first two months of life, causes cardiomyopathy, and is typically fatal in the first year of life [4]. The nonclassical infantile form of Pompe disease typically presents in the first year of life, progresses more slowly than the classical infantile onset form, and typically leads to respiratory failure without cardiomyopathy and death in later childhood. Later-onset forms of Pompe disease, with onset ranging from infantile to early adulthood, are also possible and progress at a variable rate [3].

Targeted enzyme-replacement therapy (ERT) for Pompe disease can effectively slow disease progression by providing sufficient enzyme to degrade glycogen [5]. Because ERT has the greatest benefit if started prior to the onset of symptoms [6] and individuals with Pompe disease are typically asymptomatic at birth, newborn screening for Pompe disease has been recommended by the Secretary of the U.S. Department of Health and Human Services [7] to identify children at risk for Pompe disease as early as possible.

The Missouri State Public Health Laboratory (MSPHL) initiated universal newborn screening for Pompe disease in January 2013, following guidance set by House Bill 716, the Brady Alan Cunningham Act [8]. This bill was signed into law in 2009 and mandated the expansion of newborn screening to include Pompe disease and several other lysosomal storage disorders (LSDs). This manuscript summarizes how MSPHL has refined its Pompe newborn screening protocols over the first six years in practice and highlights important findings through the follow-up program.

## 2. Materials and Methods 

**Figure 1:**
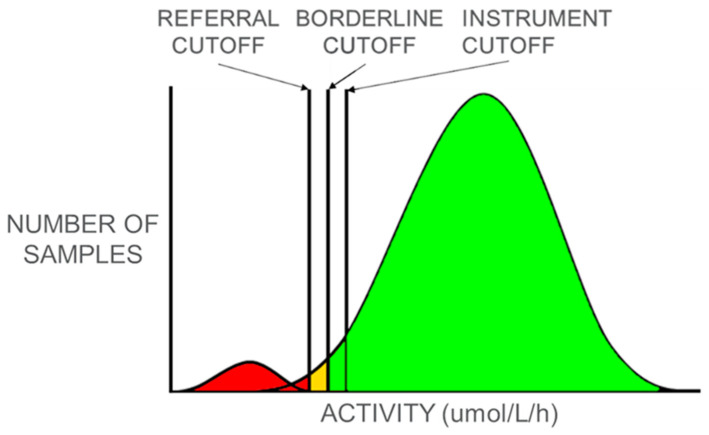
Representative depiction of the distribution of acid alpha-glucosidase (GAA) enzyme activity (low to high) in newborns. The small population to the left of the referral cutoff (depicted in red) indicates the high risk patient population that is referred for confirmatory testing. The large distribution to the right of the borderline cutoff (depicted in green) indicates the presumed normal population, who require no further action. As with many other newborn screening assays, there is an area of overlap between the affected and normal populations (indicated in yellow).

On 16 October 2012, MSPHL received a waiver from the Missouri Department of Health and Senior Services’ Institutional Review Board, as implementation of LSD screening did not meet the IRB definition of research. MSPHL utilizes digital microfluidic fluorometry (DMF) (SEEKER; Baebies, Inc., Durham, NC) for Pompe disease screening [9,10,11]. Decreased enzyme activity of acid α-glucosidase (GAA) indicates an increased risk for Pompe disease. MSPHL initially established cutoffs based on the enzyme activity of diagnostic samples from patients with Pompe disease, which were provided by the Missouri genetic referral centers. These samples included newborn and nonnewborn samples from patients with infantile Pompe as well as later-onset Pompe. As more data was collected from routine newborn screening for Pompe, cutoffs were refined to better reflect newborn enzyme activity levels. As depicted in Figure 1, samples with measured enzyme activity above the instrument cutoff are presumed to be normal and no further testing is performed. For any samples with measured activity below the instrument cutoff, testing is repeated in duplicate and the average of the three values is calculated and used to assess risk. If the average value is below the referral cutoff, the baby is referred to a genetic referral center for evaluation and diagnostic confirmatory testing. If the average value is below the borderline cutoff but above the referral cutoff, an additional newborn screening sample is requested to repeat the dried blood spot (DBS) screening test. If the average value is above the borderline cutoff, the sample is presumed normal.

The cutoffs initially established by MSPHL did not account for age of the baby at sample collection, but after gathering population data over time, it was apparent that GAA enzyme activities decreased with the age of the newborn at the time of sample collection and that age-related cutoffs should be utilized. New cutoffs for samples from newborns >14 days of age at collection were implemented in mid-2013 and further refined as more data were collected and analyzed. MSPHL also discovered that lysosomal enzyme activities were affected by seasonal variability. Enzyme activities were reduced during the summer months, causing an increase in false positive referrals. After observing this trend, MSPHL implemented cutoff adjustments between winter and summer to reduce unnecessary referrals. Because four lysosomal enzymes are measured simultaneously with the DMF method, MSPHL leverages all four lysosomal enzyme results to ascertain sample quality. If multiple lysosomal enzyme activities are below their respective cutoffs, the sample is considered to have compromised quality and an additional newborn screening sample is requested. Whereas many other screening programs base GAA cutoffs on the percent of the daily mean or median, MSPHL has found that with the relatively small number of births per day in Missouri, fixed cutoffs that can be modified seasonally are more effective.

Because the need for second-tier testing for some LSDs was unrecognized when MSPHL began screening and there were no established second-tier testing options available at the time, MSPHL did not employ second-tier testing for Pompe disease. All presumptive positive screens were referred to a genetic referral center for diagnostic testing. In Missouri, there are four such centers to accept these patient referrals, and referrals are made based on predetermined geographic boundaries. The diagnostic testing performed for a presumptive positive patient is dependent on the referred condition; for Pompe disease, this testing may include leukocyte GAA enzyme activity, creatine kinase (CK) enzyme activity, urinary glucotetrasaccharide (HEX4), targeted gene sequencing, and cardiac evaluation. Additional testing may include quantification of lactic acid dehydrogenase (LDH), CK-MB (an isoenzyme of creatine kinase that is found mostly in the heart), liver enzymes (AST and ALT), and/or brain natriuretic peptide (BNP). While confirmatory testing for Pompe disease follows a general protocol, the procedure for each patient is highly dependent on the specific clinical presentation.

The predicted onset—infantile or late—is made during the time of confirmatory testing based on biochemical test results, imaging, clinical presentation including presence of cardiomyopathy, and variant analysis. Table 1 outlines the criteria used by the Missouri follow-up program to determine disease status. These are the general guidelines; the final classification is made by the follow-up team based on evaluation of all of the clinical information. Following a confirmation of infantile onset Pompe disease, treatment with enzyme replacement therapy is typically initiated as quickly as possible; in patients with a diagnosis of later-onset Pompe, treatment with ERT is typically delayed until the onset of symptoms or laboratory results consistent with progression of disease are observed.

## 3. Results

### 3.1. Screening Results

In the first six years of LSD screening (January 2013 through December 2018), MSPHL tested approximately 467,000 newborns, of which 274 screened positive based on decreased GAA activity. Results of confirmatory testing for these specimens are presented in Table 2.

Ten newborns were found to have infantile Pompe disease, and 36 were found to have later-onset Pompe disease. Eight newborns were found to have genotypes of unknown significance (GUS), and 51 newborns that screened positive were found to have pseudodeficiency variants. The false positive rate (FPR—0.05%) and the positive predictive value (PPV—17.1%) for the Pompe assay is comparable to published prospective screening results for GAA without the use of second-tier screening [12] and for other newborn screening tests in general [13].

Each genetic referral center was contacted to request follow-up information for newborns diagnosed with Pompe disease or a genotype of unknown significance. For each case, the referral center was asked to provide variant information and biochemical or other diagnostic test results. Additionally, referral centers were surveyed with specific questions about the current developmental status of each patient remaining in active follow-up. This survey collected binary responses (e.g., improved/unchanged/worsening) for symptoms in the following categories: cardiac, myopathy/hypotonia, respiratory, feeding, hearing, overall development, and laboratory test results (normal/abnormal).

Data from confirmatory testing for newborns confirmed with Pompe disease (infantile and later-onset) as well as newborns with genotypes of unknown significance was compiled (Table 3).

### 3.2. Confirmed Positive Pompe Patients

#### 3.2.1. Infantile Onset

Ten newborns were found to have infantile onset Pompe disease, with seven considered “classical” and three considered “nonclassical”. Classical infantile Pompe is differentiated from nonclassical infantile Pompe by the presence of hypertrophic cardiomyopathy at birth. Of the classical infantile onset patients, all seven received testing for urine HEX4 and CK. Six (86%) had elevated HEX4 and seven (100%) had elevated CK activity. Six cases received a cardiac workup including EKG and echocardiogram; all six cases (100%) showed evidence of cardiomyopathy. The seventh case that did not receive a cardiac workup was diagnosed via amniocentesis, and had extremely elevated HEX4 and CK levels. All seven newborns began treatment with enzyme replacement therapy (ERT) at ages ranging from four days to one month. One of these patients was CRIM-negative and thus underwent immunosuppressive therapy prior to receiving ERT.

Three newborns were diagnosed with nonclassical infantile onset Pompe disease. Two of the three newborns were from the same family (separate births); both newborns were compound heterozygotes for the severe c.525DelT variant and the common later-onset c.-32-13T>G variant. These two newborns had normal HEX4 levels but mildly elevated CK. While the first newborn’s HEX4 and cardiac workup were normal, the patient’s liver enzymes (AST and ALT) were abnormal, CK-MB and LDH were elevated, and the newborn was failing to thrive. This newborn was initiated on enzyme replacement therapy at 29 days of age. The second newborn also had normal HEX4 but showed mild concentric left ventricular hypertrophy and elevated LDH. This newborn began treatment with ERT at 1 month of age. The third newborn with nonclassical infantile onset Pompe disease had significantly elevated HEX4 and CK. This newborn was compound heterozygous for the c.2560C>T variant, which is commonly associated with classical infantile onset Pompe disease and the c.2236T>C variant, which is a less common missense variant. This newborn began ERT at 1 month of age.

#### 3.2.2. Later-Onset

Through screening, 36 newborns were identified and subsequently diagnosed with later-onset Pompe disease. Of these, 35 newborns received tests for HEX4 only (*n* = 7), CK only (*n* = 9), or both (*n* = 19). All newborns that received testing had normal results for HEX4 and six (21%) had mildly elevated CK. Ten of the newborns were found to have abnormal results for some combination of liver enzymes, LDH, CK-MB, and/or BNP.

Sixteen newborns received cardiac testing including a combination of chest x-ray, EKG, and echocardiogram—of which 15 were within normal limits. The other newborn in this group had an abnormal ECG with concern for right ventricular or biventricular hypertrophy. This newborn was compound heterozygous for the c.2560C>T variant and the c.-32-13T>G variant. During follow-up testing, this newborn exhibited worsening cardiomyopathy and hypotonia as well as abnormal labs; the newborn began ERT at 13 months of age.

#### 3.2.3. Genotypes of Unknown Significance

Eight newborns were found to have genotypes of unknown significance. All eight received testing for either HEX4 only (*n* = 1), CK only (*n* = 3), or both (*n* = 4); all results were normal. Additionally, three of the newborns received an EKG and echocardiogram; these results were also normal. The genotypes of these eight patients were all different, and four of the eight had at least three detected variants. Two of these newborns remain in active follow-up and neither has begun to show clinical manifestations of Pompe disease. For two others, follow-up was deemed unnecessary unless clinical concerns arose, and the remaining four cases were lost to follow-up.

#### 3.2.4. Pseudodeficiencies

Through screening, 53 newborns were found to have GAA pseudodeficiency. Three common GAA pseudodeficiency variants were represented in the Missouri population: c.1726G>A, c.2065G>A, and c.271G>A, including the common c.1726G>A/c.2065G>A haplotype. In total, the incidence of pseudodeficiency homozygotes or compound heterozygotes was 1:8811 (0.01%).

#### 3.2.5. Other Results

Sixty five newborns were found through confirmatory testing to be heterozygous for a pathogenic variant, likely pathogenic variant, or variant of unknown significance. Additionally, 97 newborns were classified as normal due to normal confirmatory enzyme activities.

### 3.3. Current Follow-up Status

Of the 54 newborns identified with infantile onset or later-onset Pompe disease, or a genotype of unknown significance, 59% (32/54) remain in active follow-up with the genetic referral centers. This includes 7/10 infantile onset cases, 23/36 later-onset cases, and 2/8 with genotypes of unknown significance.

All ten patients with infantile onset Pompe disease—seven classical and three nonclassical—initiated enzyme replacement therapy at ages between four days and one month. Four of the classical infantile onset cases remain in active follow-up (two of the patients have moved out of the state and one is deceased). Cardiac symptoms (hypertrophic cardiomyopathy) have improved in all four active cases (4/4). In three of the cases, myopathy/hypotonia, growth, respiratory symptoms, hearing, feeding, and overall development have improved or remained unchanged since the initiation of ERT. In the remaining case, in which the patient has been receiving ERT for approximately 5.5 years, myopathy and hypotonia have worsened, hearing loss has occurred, and overall development has slowed.

All three of the newborns with nonclassical infantile onset remain in active follow-up. Cardiac symptoms, hypotonia, respiratory, hearing, feeding, and development status are unchanged in all three cases. In one case, ERT was discontinued within the first year of life due to infusion-related reactions. A desensitization protocol was attempted and unsuccessful. This patient is still followed closely and continues to have normal labs, normal growth, and no pulmonary concerns.

Of the 23 later-onset cases that are still in active follow-up, the status of 20/23 is unchanged and ERT has not been administered. In one case, cardiac symptoms improved without the aid of ERT; in another case, both cardiac symptoms and myopathy improved without ERT. In the final case, hypotonia worsened on follow-up and labs were abnormal, which resulted in the introduction of ERT at 13 months of age.

## 4. Discussion

### 4.1. Screening Results

Through nearly 6 years of prospective testing, Missouri screened approximately 467,000 newborns for GAA activity; as a result of this screening, 46 newborns were diagnosed with Pompe disease, including 10 with infantile onset Pompe disease. From the evidence report compiled in 2013 by the Condition Review Workgroup of the Advisory Committee on Heritable Diseases in Newborn and Children (ACHDNC), the incidence of Pompe disease in the United States was estimated at 1:28,000, with approximately 28% of the cases (1:100,000) presenting in the first 12 months of life (infantile onset) [4]. Missouri’s screening program identified a higher than expected overall rate of Pompe disease (1:10,152) and infantile Pompe disease (1:46,700), with a slightly lower than expected percentage of cases diagnosed with infantile onset (22%).

**Figure 2:**
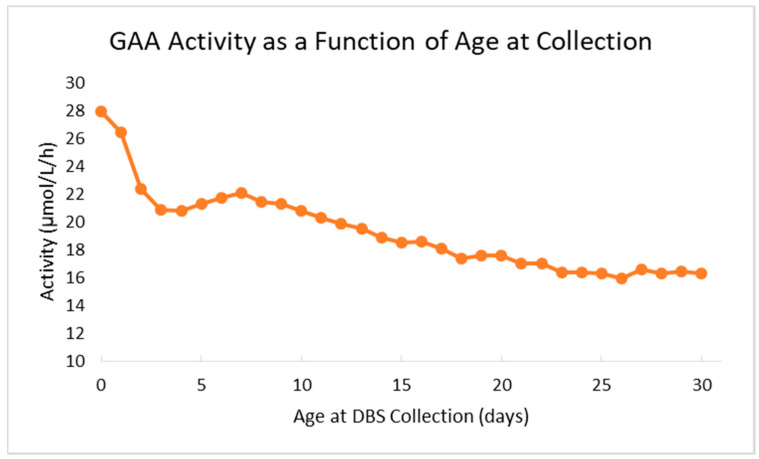
GAA enzyme activity decreases during the first 30 days of life. GAA enzyme activity data (Y axis) from this chart includes average screening results from 338,743 full-term newborns (>38 weeks gestational age) prospectively screened at MSPHL. Age at DBS sample collection (X axis) was rounded down to the nearest full day.

The Missouri newborn screening laboratory overcame several challenges during the first months of screening for Pompe. As the first U.S. state to screen for Pompe, there was limited data available for the laboratory to reference when determining preliminary cutoffs, specifically for demographic variables. For example, the laboratory implemented age-related cutoffs for GAA activity after approximately four months of live screening when it was observed that median GAA activity decreases by more than 30% from birth to the 14th day of life. The decrease in median enzyme activity as a function of age at sample collection is illustrated in Figure 2. After implementation of age-related thresholds, the retest and false positive rates for the assay both decreased.

Similarly, the laboratory began to utilize the activity of all tested lysosomal enzymes as a measure of sample quality. This was beneficial with leukodepleted samples; since lysosomal enzymes in blood are predominantly present in white cells, activity of all lysosomal enzymes can be decreased in leukodepleted samples. Evaluation of all lysosomal enzymes is also useful during periods of high temperature and humidity. During the summer months, lysosomal enzymes may be denatured during extended exposure to elevated heat and humidity. Beginning the first summer following the start of screening, MSPHL has used all lysosomal enzyme activities as an indicator of sample quality and considers results that were abnormal for multiple lysosomal enzymes to be inconclusive, thereby requiring only a repeat/second newborn screen. The laboratory also adjusts the cutoffs during the summer months to account for the decrease in activity.

Comparison of HEX4 and CK, two diagnostic tests that are completed during the Pompe follow-up protocol, shows a correlation between disease severity and HEX4 and CK levels. The classical infantile onset patients all displayed elevated HEX4 and CK, while the nonclassical infantile and later-onset patients had results within the normal limits or mildly elevated results. Although CK activity for the nonclassical infantile cases was only mildly elevated, these cases were significantly elevated (median = 416) relative to the later-onset group (median = 126).

### 4.2. Variant Frequencies

#### 4.2.1. Infantile and Later-Onset Variants

Of the 10 newborns diagnosed with infantile onset Pompe through screening, a total of 13 different variants were identified. Specific variant information is presented in Table 4. The c.525DelT variant was the most common infantile onset variant in this cohort, as was found in 3/10 cases, including two newborn family members prospectively identified through screening. The c.1447G>A, c.1802C>T, c.2560C>T, c.-32-13T>G, and exon 18 deletions were found in two patients each.

Of the later-onset patients, 36 different variants were detected. The most common variant detected was the c.-32-13T>G variant, which was found in 66.7% (24/36) patients, including 10 homozygotes. The c.841C>T variant was also found as part of a compound heterozygote in four different patients.

#### 4.2.2. Pseudodeficiency

The frequency of pseudodeficiency variants was also evaluated. Of the 53 cases classified as pseudodeficiencies, variant information was available for 49 newborns. Of these 49 newborns, 48 (98%) had the common c.1726G>A/c.2065G>A pseudodeficiency haplotype. Two newborns also possessed the c.271G>A pseudodeficiency variant; one newborn was homozygous for this allele, and one newborn possessed this allele as a compound heterozygote with the c.1726G>A/c.2065G>A haplotype. When evaluating the ethnicities of the pseudodeficiency cases, 43 (88%) of the 49 cases with available variant information reported the newborn’s ethnicity on the sample collection form as either “Asian”, “Pacific Islander”, or multiethnic including at least one of those two groups. These pseudodeficiency variants have previously been reported at high prevalence in the Asian population [14,15], and the results of screening in Missouri indicate that the vast majority of GAA pseudodeficiency cases are of Asian ethnicity. Based on the 2010 U.S. Census [16], 2.0% of Missouri’s population is of Asian ethnicity, which is significantly below the U.S. average of 5.6%; other U.S. states or territories with a higher population proportion of Asian ethnicity may encounter higher rates of pseudodeficiency.

### 4.3. Current Follow-up Status

Fifty nine percent of newborns with Pompe disease or genotypes of unknown significance have maintained active follow-up after the initial diagnosis. All infantile onset patients remain in active follow-up with the exception of two families that no longer reside in the state; however, the proportion of later-onset patients and patients with genotypes of unknown significance that maintain active follow-up is far lower (23 of 36 later-onset, 64%, remain active; 2 of 8, 25%, GUS remain active).

Of the 10 infantile onset cases diagnosed, all 10 started enzyme replacement therapy. Nine of the infantile cases were CRIM-positive. Eight of ten have improved or unchanged symptoms, with one case of worsening hypotonia after more than 5 years on ERT, and another discontinuing ERT after less than one year following development of infusion-related reactions. Both of the newborns with worsening symptoms or adverse reactions were CRIM-positive. The CRIM-negative newborn has moved out of state and is no longer in active follow up in Missouri. One of the later-onset cases has developed symptoms consistent with Pompe disease and has initiated treatment; as this patient was 13 months of age at initiation of treatment, it reinforces that patients with later-onset Pompe disease may develop symptoms and require intervention with ERT in early childhood.

## 5. Conclusions

The Missouri newborn screening program continues to operate the longest continuous Pompe screening program in the United States and has screened approximately 467,000 newborns during its first six years of screening. Pompe screening has been very successful as 46 patients (approximately 1:10,000) have been diagnosed with Pompe disease, including 10 children (1:46,700) with infantile onset disorder. MSPHL also detected several common pseudodeficiency variants through screening, which caused an increased false positive rate. Implementation of second-tier testing would improve the FPR and PPV, as these pseudodeficiency variants are prevalent in the state’s population; Missouri is currently evaluating options to implement second-tier testing for Pompe as well as use of postanalytical tools. Through analysis of screening results, the laboratory found that GAA activity decreases from birth to the 14th day of life and requires age-related cutoffs for the most appropriate risk assessment. MSPHL also found that testing for multiple lysosomal enzymes can aid in the determination of sample quality, as the lysosomal enzyme activities can be decreased in leukodepleted samples or in samples that are exposed to elevated heat and humidity.

To date, 11 patients (10 with infantile onset, 1 with later-onset) have begun treatment with enzyme replacement therapy through the screening and follow-up program. Early initiation of ERT has led to normal development and cardiac improvement for the majority of the infantile onset Pompe disease cases, and only one patient has discontinued ERT due to infusion-related reactions. Additionally, the presence of a patient with diagnosed later-onset disease that has already begun ERT offers a case study that later-onset disease may still present in infancy or early childhood.

## Figures and Tables

**Table 1:** Missouri Newborn Screening Follow-Up Criteria for Pompe Disease.

Newborn Assessment	Classical Infantile	NonClassical Infantile	Later Onset	Genotype of Unknown Significance	Pseudodeficiency	Carrier
GAA enzyme activity	Absent or within affected range	Within affected range	Decreased	Decreased	Decreased	Decreased or normal
HEX4	Elevated	Elevated or WNL	WNL	WNL	WNL	WNL
Creatine Kinase (& other labs as indicated)	Elevated	Elevated or WNL	WNL	WNL	WNL	WNL
Chest x-ray, EKG, Echo	Abnormal	Mild abnormalities or WNL	WNL	WNL	WNL	WNL
Variant analysis	-Two pathogenic variants -One pathogenic variant and one or more VUS -Two VUS	-Two pathogenic variants -One pathogenic variant and one or more VUS -Two VUS	-Two pathogenic variants -One pathogenic variant and one or more VUS -Two VUS	-One infantile variant and one or more VUS -One late onset variant and one or more VUS -Two or more VUS	-Two pseudodeficiency alleles	-One pathogenic variant -May or may not be in combination with pseudo alleles
Clinical presentation	Muscle weakness, poor muscle tone, feeding issues, cardio-myopathy present	Muscle weakness or WNL	WNL at birth	WNL at birth	WNL at birth	WNL at birth

Abbreviations: WNL = within normal limits; VUS = variant(s) of unknown significance.

**Table 2:** Results of Confirmatory Pompe Testing.

Total Screened	~467,000
Screen Positives	274
Confirmed Disorders	46
Infantile Onset Pompe Disease	10
Later-onset Pompe Disease	36
Genotypes of Unknown Significance	8
Pseudodeficiencies	53
Carriers	65
Normal	97
Lost to Follow-up	5
Positive Predictive Value (PPV)	17.1%
False Positive Rate (FPR)	0.05%

**Table 3:** Confirmatory Test Results for Patients with Pompe Disease or Genotypes of Unknown Significance (GUS).

Disease Classification	HEX4 (nmol/mol Creatinine)			Creatine Kinase (U/L)		
	*n* (Data Reported)	Median	Range	*n* (Data Reported)	Median	Range
Classical Infantile	7 (7)	22.7	13.4–38.6	7 (7)	662	466–3537
Nonclassical Infantile	3 (3)	5	3.7–25.2	3 (2)	416	398–435
Later-onset	36 (26)	4.65	2.3–12.3	36 (25)	127	50–466
GUS	8 (5)	6.6	2.3–7	8 (7)	87	71–203
Normal Range	<20 nmol/mol creatinine			<305 U/L		

**Table 4:** Variants Identified in Infantile Pompe Disease Patients through Prospective Screening.

Diagnosis	Variants
Classical Infantile	c.1548G>A/del exon 18
Classical Infantile	c.1447G>A/c.2560C>T
Classical Infantile	c.670C>T/c.2481+31Del
Classical Infantile	c.525DelT/c.1447G>A
Classical Infantile	c.1827C>G/c.2662G>T
Classical Infantile	c.1802C>T/c.1802C>T
Classical Infantile	c.947A>G/del exon 18
Nonclassical Infantile	c.-32-13T>G/c.525DelT
Nonclassical Infantile	c.-32-13T>G/c.525DelT
Nonclassical Infantile	c.2560C>T/c.2236T>C
